# *Arabidopsis* LBP/BPI related-1 and -2 bind to LPS directly and regulate *PR1* expression

**DOI:** 10.1038/srep27527

**Published:** 2016-06-08

**Authors:** Sayaka Iizasa, Ei’ichi Iizasa, Sawako Matsuzaki, Hiroyuki Tanaka, Yutaka Kodama, Keiichi Watanabe, Yukio Nagano

**Affiliations:** 1 Analytical Research Center for Experimental Sciences, Saga University, 1 Honjo-machi, Saga 840-8502, Japan; 2 Department of Biological Science and Technology, The United Graduate School of Agricultural Sciences, Kagoshima University, 1-21-24 Korimoto, Kagoshima 890-8580, Japan; 3 Department of Immunology, Graduate School of Medical and Dental Sciences, Kagoshima University, 8-35-1 Sakuragaoka, Kagoshima 890-8544, Japan; 4 Department of Applied Biochemistry and Food Science, Faculty of Agriculture, Saga University, 1 Honjo-machi, Saga 840-8502, Japan; 5 Center for Bioscience Research and Education, Utsunomiya University, Tochigi 321-8505, Japan

## Abstract

Lipopolysaccharide (LPS) is a major constituent of the outer membrane of Gram-negative bacteria and acts as a pathogen-associated molecular pattern that triggers immune responses in both plants and animals. LPS-binding protein (LBP) and bactericidal/permeability-increasing protein (BPI), which bind to LPS and play important roles in immunity of mammals, have been well studied. However, the molecule contributing to LPS binding in plants is mostly unknown. The *Arabidopsis* genome carries two genes encoding LBP/BPI-related proteins which we designated as *AtLBP/BPI related-1* (*AtLBR-1*) and *AtLBP/BPI related-2* (*AtLBR-2*). We found that their N-terminal domains were co-purified with cell wall-derived LPS when expressed in *E. coli*. Since this finding implied the direct binding of AtLBRs to LPS, we also confirmed binding by using LPS-free AtLBRs and purified LPS. AtLBRs directly bind to both rough and smooth types of LPS. We also demonstrated that LPS-treated *atlbr* mutant *Arabidopsis* exhibit a significant delay of induction of defence-related gene *pathogenesis-related 1* (*PR1*) but no other *PR* genes. Furthermore, LPS-treated *atlbr* mutants showed defects in reactive oxygen species (ROS) generation. These results demonstrate that, as well as LBP and BPI of mammals, AtLBRs also play an important role in the LPS-induced immune response of plants.

Plants detect pathogen invasions by recognition of pathogen-associated molecular patterns (PAMPs). PAMP perception induces various defence responses. Lipopolysaccharide (LPS), a primary constituent of the outer membrane of Gram-negative bacteria, is one of the most studied PAMPs. LPS causes defence responses such as generation of nitrogen oxide (NO) and reactive oxygen species (ROS) in *Arabidopsis*, tobacco, and rice suspension cells^1^,^2^,^3^. LPS also induces expression of *pathogenesis-related* (*PR*) genes, which are up-regulated in pathological or stressful situations, in *Arabidopsis* leaves^1^. In addition, LPS induces stomatal closure and NO production in guard cells^3^. Further, investigation of the LPS recognition mechanism is at the forefront in plant innate immunity studies. Recently, Ranf *et al.* identified that the bulb-type lectin S-domain-1 receptor-like kinase LORE (lipooligosaccharide-specific reduced elicitation) is required for sensing of LPS from *Pseudomonas* and *Xanthomonas* species^4^. However, it is still unclear which molecule(s), including LORE, can directly bind to LPS. Thus, the identification of these molecule(s) will enhance our understanding of the overall LPS recognition system.

In mammals, there are two well-studied proteins that directly bind to LPS, LPS-binding protein (LBP) and bactericidal/permeability-increasing protein (BPI). Human LBP (hLBP) and human BPI (hBPI) structurally resemble each other with 45% amino acid sequence identity. Both proteins play important roles in the regulation of defence responses against LPS. Mammalian LPS recognition is orchestrated by several LPS binding proteins, including LBP, BPI, and a membrane protein CD14, which transfers LPS to a mammalian LPS receptor complex TLR4/MD-2^5^,^6^. LBP, a serum glycoprotein produced principally by hepatocytes, is critical to rapid and effective signal transduction for induction of proinflammatory cytokines, because it facilitates the transfer of LPS to CD14, then to TLR4/MD-2. In addition, BPI, which is a glycoprotein purified from granules of neutrophils, also binds to LPS with higher affinity than LBP^5^. The binding of BPI to LPS increases the permeability of the bacterial membranes and opsonizes bacteria to enhance phagocytosis by neutrophils^5^. Another important aspect of BPI is the attenuation of the LPS-induced inflammatory response by competitive inhibition against LBP^5^.

LBP and BPI belong to a protein family called the LBP/BPI/PLUNC (palate, lung, and nasal epithelial clone) superfamily^7^. PLUNC protein, an abundant secretory product in human nasal lavage fluid, also can bind to LPS and has been shown to suppress the growth of bacteria^8^,^9^. Interestingly, LBP/BPI/PLUNC superfamily proteins have been identified in various species including chicken, fish, and oyster^10^,^11^,^12^,^13^,^14^,^15^. This superfamily can be divided into two subfamilies; LBP/BPI and PLUNC. Ovocalyxin-36 (OCX-36) is an abundant eggshell protein of chicken which is related to the PLUNC subfamily. OCX-36 binds to LPS and shows inhibitory activity against growth of *Staphylococcus aureus*^16^. LBP/BPI subfamily proteins of oyster *Crassostrea gigas* (Cg-BPI1 and Cg-BPI2) also display LPS binding and bactericidal activities^15^,^17^. Expression of LBP/BPI subfamily genes was induced by bacterial challenge or LPS treatment in various fish such as rainbow trout, Atlantic cod, carp, and ayu^11^,^12^,^13^,^14^. However, LBP/BPI/PLUNC superfamily proteins have not been characterised in plants. The fact that members of both subfamilies (i.e., hLBP, hBPI, OCX-36 and Cg-BPI) bind to LPS and participate in innate immune responses against potential bacterial invasion motivated the current study to characterize plant proteins belonging to the LBP/BPI/PLUNC superfamily.

Here, we characterised two genes of *Arabidopsis*, *AtLBP/BPI related-1* (*AtLBR-1*) and *AtLBP/BPI related-2* (*AtLBR-2*), which belong to the LBP/BPI subfamily rather than the PLUNC subfamily. Because many LBP/BPI/PLUNC superfamily proteins were characterised by their LPS binding ability, we studied whether AtLBRs can bind to LPS. As the results, the recombinant N-terminal domain of AtLBRs is found to bind to LPS directly. In addition, LPS-treated *atlbr* mutants showed the defect in immune responses, such as *PR1* gene expression and ROS production. Altogether, these results demonstrate the biological importance of LBRs for induction of LPS-triggered defence responses in plants and the functional similarities among LBP/BPI subfamily from various organisms.

## Results

### Identification of LBP/BPI-related proteins in *Arabidopsis* genome

**Figure 1:**
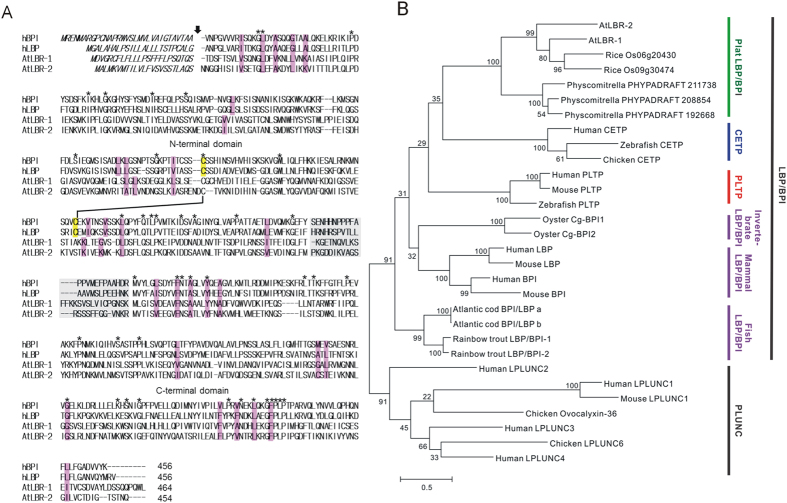
Sequence homologies and phylogenetic analysis of AtLBRs. (**A**) Multiple alignment of hBPI, hLBP, AtLBR-1, and AtLBR-2. Alignment was performed with Clustal-W. Amino acid numbers refer to the mature proteins. Conserved residues are indicated by asterisks. An arrow indicates the putative cleavage site by the signal peptidase. Conserved cysteines forming the single disulphide bond in hBPI^14^ are highlighted in yellow. The residues constituting the apolar binding pockets are highlighted in pink. Proline-rich central domains characterized for hBPI as well as the corresponding sequences in hLBP and AtLBRs are highlighted in grey. (**B**) Phylogenetic analysis of the LBP/BPI/PLUNC superfamily. The phylogenetic tree was generated by the maximum-likelihood method using MEGA6.06. Bootstrap values (%) were estimated by 1000 replications and are presented at each branch point. Protein sequences were downloaded from NCBI: AtLBR-1 (NM_100375), AtLBR-2 (NM_112918), human CETP (NM_000078), chicken CETP (NM_001034814), zebrafish CETP (BC085584), human PLTP (NM_006227), mouse PLTP (BC003782), zebrafish PLTP (NM_001003519), Cg-BPI1 (FJ669301), Cg-BPI2 (HM992925), human LBP (M35533), mouse LBP (NM_008489), human BPI (DQ414688), mouse BPI (NM_177850), Atlantic cod LBP/BPI a (AY102628), Atlantic cod LBP/BPI b (AY102629), rainbow trout LBP/BPI-1 (NM_001124585), rainbow trout LBP/BPI-2 (NM_001124198), human LPLUNC1 (NM_033197), human LPLUNC2 (NM_025227), human LPLUNC3 (NM_182658), human LPLUNC4 (NM_182519), mouse LPLUNC1 (NM_001012392), chicken LPLUNC6 (XM_417463), and chicken ovocalyxin-36 (NM_001030861). Protein sequences were downloaded from Phytozome: Os09g30474 (LOC_Os09g30474), Os06g20430 (LOC_Os06g20430), *Physcomitrella* PHYPADRAFT 211738 (Pp1s73_8V6), *Physcomitrella* PHYPADRAFT 208854 (Pp1s49_176V6), and *Physcomitrella* PHYPADRAFT 192668 (Pp1s184_35V6).

To identify putative LBP/BPI-related genes in *Arabidopsis*, a BLAST search was performed using the hLBP amino acid sequence as a query against the *Arabidopsis* genome sequence. As a result, two candidates were found. We termed these proteins AtLBR-1 (*At1g04970*) and AtLBR-2 (*At3g20270*). AtLBR-1 and AtLBR-2 consist of 488 and 477 amino acids including signal sequences, respectively. Putative mature proteins of AtLBR-1 and AtLBR-2 have calculated molecular masses of 50.7 kDa and 49.8 kDa, respectively. They share 41% amino acid sequence identity to each other. Both proteins display 42–46% similarity and 23–25% identity to the amino acids of hLBP and hBPI, respectively. A multiple alignment of LBP/BPI proteins showed that the residues determining backbone flexibility (Gly) or rigidity (Pro) are highly conserved (Fig. 1A). In BPI, two apolar binding pockets, located in each N- and C-terminal domain, are thought to play a role in binding of LPS acyl chains^18^. The hydrophobic residues constituting the polar binding pockets are also highly conserved in both AtLBR-1 and AtLBR-2 (Fig. 1A). In contrast, a pair of cysteine residues, which form an intramolecular disulphide bond in hBPI^18^, is not found in the AtLBR N-terminal domains.

Phylogenetic trees were constructed using the maximum-likelihood method with amino acid sequences of AtLBR proteins and members of the LBP/BPI/PLUNC superfamily (Fig. 1B). The tree was divided into two major branches, LBP/BPI and PLUNC, as shown previously^7^. AtLBRs were clearly classified as a LBP/BPI subfamily. Plant members of the LBP/BPI subfamily are clustered together and are clearly distinct from the animal sub-branch of LBP/BPI subfamily. Although AtLBRs have sequences related to LBP/BPI proteins of animals, they evolved independently from animal ones.

### Recombinant AtLBR-Ns were co-purified with LPS derived from expression host *E coli*

**Figure 2:**
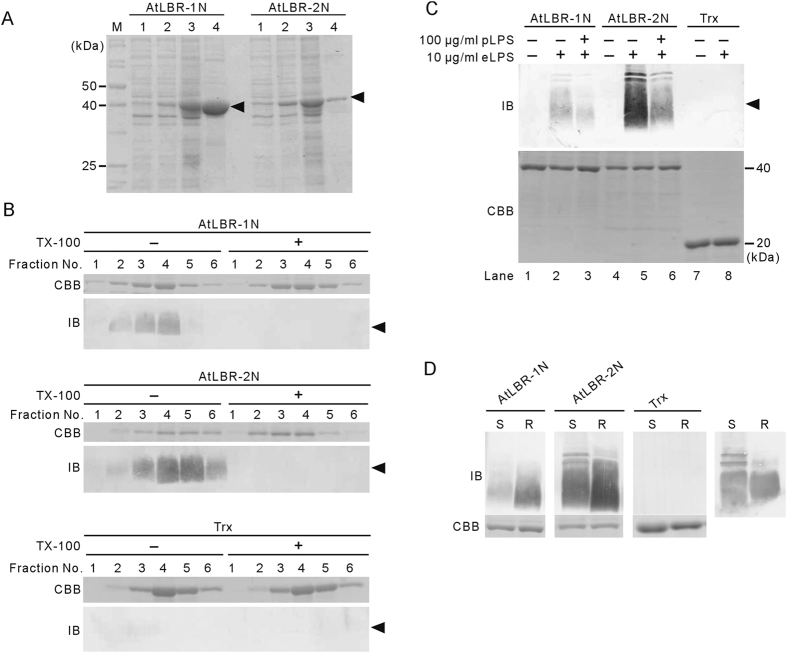
Direct binding of AtLBR-1N and AtLBR-2N to LPS. (**A**) AtLBR-1N and AtLBR-2N were expressed in *E. coli* and purified by Ni-affinity chromatography (arrowhead). Lane 1: bacterial lysate before induction, Lane 2: bacterial lysate after IPTG-induction, Lane 3: soluble fractions, and Lane 4: purified fractions by Ni-affinity chromatography were analysed by SDS-PAGE and CBB staining. (**B**) Recombinant AtLBR-Ns and control Trx were purified by Ni-affinity column in the absence or presence of 0.5% Triton X-100. Eluates were analysed by SDS-PAGE (12% gel) followed by CBB staining (upper panels). LPS in the fractions were detected by immunoblotting (18% gel) using an anti-eLPS antibody (arrowhead, lower panels). (**C**) 10 μg/ml LPS-free recombinant proteins were incubated with 10 μg/ml purified eLPS with or without 100 μg/ml purified pLPS. The LPS-protein complexes were purified using His Spin Trap columns and recombinant proteins were analysed by SDS-PAGE (12% gel) followed by CBB staining (lower panels). eLPS bound to the recombinant protein was detected by anti-eLPS immunoblotting (18% gel, arrowhead) (upper panels). (**D**) 10 μg/ml LPS-free recombinant proteins were incubated with 10 μg/ml smooth (S) and rough (R) LPS from *E. coli*. The binding of the proteins to S and R LPS was examined as above. S and R LPS (100 ng per lane) were used as controls (right panel).

Because it has been reported that the recombinant N-terminal domain of hLBP was co-purified with LPS derived from expression host *E. coli*^19^, we investigated if AtLBRs could be co-purified with LPS. We prepared a recombinant protein of AtLBR-1 containing 215 N-terminal residues (AtLBR-1N) and of AtLBR-2 containing 214 N-terminal residues (AtLBR-2N) as thioredoxin (Trx) and His-tag fusion protein. AtLBR-Ns were successfully expressed in *E. coli* and purified by Ni-affinity chromatography (Fig. 2A). A considerable amount of LPS was detected in the eluted fractions of both AtLBR-Ns; the elution pattern of LPS completely matched those of the recombinant proteins (Fig. 2B, top and middle panels, left side). In contrast, LPS was undetectable in the eluted fractions of the negative control Trx (Fig. 2B, bottom panel, left side). These results suggest that the AtLBR-Ns bind to LPS. In addition, LPS could be removed from AtLBR-Ns by Triton X-100 as previously reported in the hLBP study (Fig. 2B, right side)^19^.

### Recombinant AtLBR-Ns directly bind to purified LPS

We examined whether AtLBR-Ns directly bind to purified LPS by using an LPS-binding assay reported previously^19^. LPS-free recombinant proteins were incubated with *E. coli* LPS (eLPS) and were trapped on a Ni-resin spin column. After washing, bound LPS was detected by immunoblotting (Fig. 2C). AtLBR-2N showed strong binding to eLPS, while eLPS binding to AtLBR-1N was relatively weak (Fig. 2C, lane 2, 5). These results demonstrate the direct binding between eLPS and AtLBR-Ns. Furthermore, the binding affinity of AtLBR-2N for eLPS seems higher than that of AtLBR-1N. In addition, we also examined whether AtLBRs bind to LPS from bacteria other than *E. coli*. *Pseudomonas aeruginosa* is an opportunistic bacterium found ubiquitously in the environment and infects many organisms from plants to humans^20^,^21^. *P. aeruginosa* LPS (pLPS) induces NO bursts and ROS production in *Arabidopsis* suspension cells^1^,^2^. Because the anti-LPS antibody cannot detect pLPS, we examined the binding of AtLBR-Ns to pLPS by a competition assay. A ten-fold amount of pLPS inhibited the binding of eLPS to AtLBR-Ns (Fig. 2C, lane 3, 6). These results show that AtLBRs interact with not only LPS from *E. coli* but also other Gram-negative bacteria such as *P. aeruginosa*.

### Recombinant AtLBR-Ns bind to both smooth and rough LPS

Gram-negative bacteria have either smooth or rough LPS. Smooth type LPS is composed of O-antigen repeats, a core oligosaccharide, and lipid A, while rough type LPS lacks the O-antigen repeats. Recombinant hLBP including the N-terminal domain binds equally well to both smooth and rough LPS^19^. Although hBPI also binds to both types of LPS directly, it has more potent bactericidal activity against bacteria containing rough type LPS^22^,^23^. Therefore, we examined the binding ability of AtLBR-Ns to smooth and rough LPS. The anti-LPS antibody could detect both types of LPS at the same level (Fig. 2D, right panel). Like hLBP and hBPI, AtLBR-Ns also bound to both smooth and rough LPS (Fig. 2D). However, both AtLBR-Ns could bind to rough type LPS with higher affinity than to smooth type. Furthermore, the binding affinity of AtLBR-2N for both LPS seemed higher than that of AtLBR-1N, which is consistent with the above data (Fig. 2C).

### Cellular localisation and expression pattern of AtLBRs

**Figure 3:**
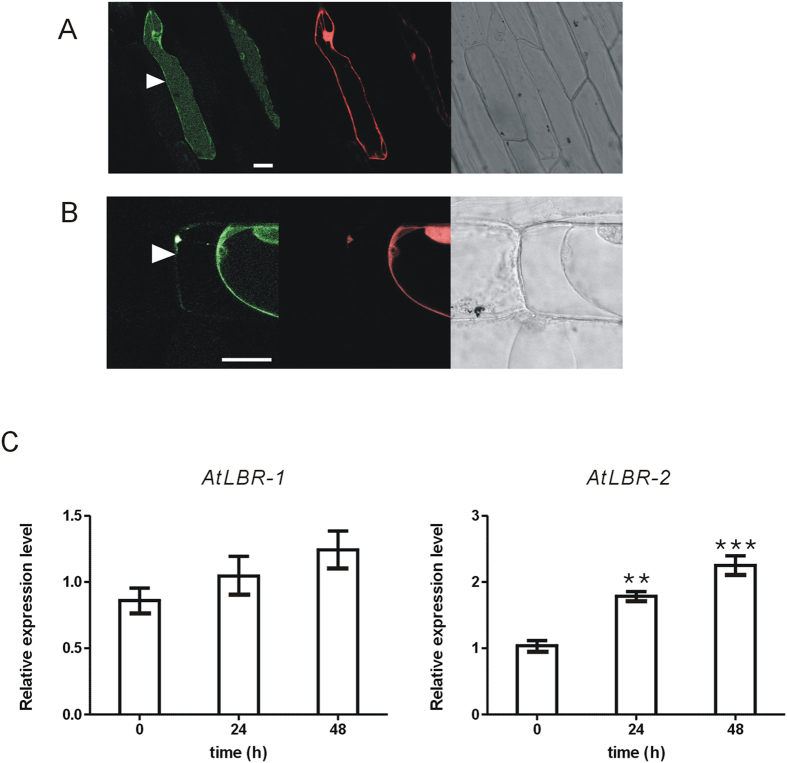
Cellular localisation and expression pattern of AtLBRs. The AtLBR-1-sfGFP (**A**) and AtLBR-2-sfGFP (**B**) fusion proteins were transiently expressed in onion epidermal cells by particle bombardment, and observed with a confocal laser-scanning microscope. Co-bombarded DsRed-monomer was used as a cytosolic marker protein. The arrowhead indicates a plasmolysed cell (**A**) and the apoplastic region (**B**). Scale bar, 50 μm. (**C**) *AtLBRs* mRNA levels in WT *Arabidopsis* seedlings treated with 100 μg/ml pLPS for 24 or 48 h were detected by qRT-PCR. Mean expression values were calculated from the results of three independent experiments. Means ± standard errors are presented. Significant differences among means compared to 0 h treated *Arabidopsis* seedlings were determined by one-way ANOVA followed by *post hoc* Tukey’s multiple comparison test. **P < 0.01, ***P < 0.001.

In many cases, plant pathogens proliferate in the leaf apoplast, thus a number of PAMPs and other extracellular elicitors are also present in the apoplast^24^. Therefore, the apoplast is an important place for plant–pathogen interactions to occur that exclude pathogens. It contains antimicrobial proteins such as proteases, glucanases, chitinases, and chitin-binding proteins. Furthermore, apoplastic ROS generation can function as signalling molecules for plant innate immune responses^24^. Therefore, we analysed the subcellular localisation of AtLBRs to determine whether or not AtLBRs are expressed in the apoplast. The AtLBR-super folder GFP (sfGFP) construct was introduced into onion epidermal cells by particle bombardment. Transient expression of the fusion protein was observed with a confocal laser-scanning microscope. To observe the apoplastic region, 20% sucrose was used to induce plasmolysis before observation. AtLBR-1-sfGFP and AtLBR-2-sfGFP proteins were co-localised with DsRed protein, which is a cytosolic marker; the fluorescent signals were also detected in vacuolar regions (Fig. 3A,B). When the regions of plasmolysis were observed, AtLBR-2-sfGFP, but not AtLBR-1-sfGFP, was detected in the apoplastic region (Fig. 3B). This result suggests that AtLBR-1 and AtLBR-2 may function specifically in different expression sites, and AtLBR-2 may play an important role in LPS-induced innate immune responses in the apoplast. As AtLBRs can bind to LPS directly *in vitro*, we investigated the possible involvement of AtLBRs in the LPS-induced innate immune responses *in vivo*. First, we analysed gene expression of *AtLBRs* in *Arabidopsis* seedlings (Fig. 3C). After whole seedlings were treated with pLPS for 24 and 48 h, *AtLBR* expression was analysed by qRT-PCR. The amount of *AtLBR-1* mRNA was not affected by pLPS treatment. In contrast, *AtLBR-2* mRNA level was increased by pLPS treatment (Fig. 3C).

### *Arabidopsis atlbr* mutants showed defects in up-regulation of *pathogenesis-related 1* (*PR1*) gene expression and ROS generation induced by LPS

**Figure 4:**
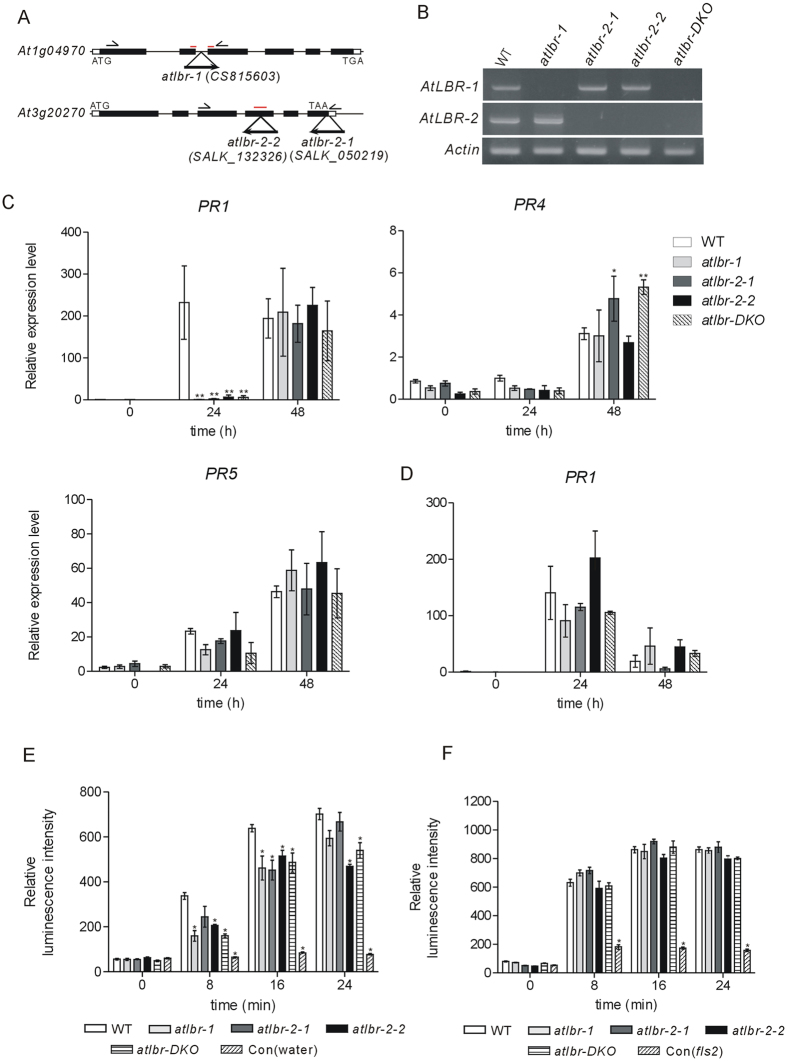
Defect in LPS-induced *PR1* gene expression and ROS generation in *atlbr* mutants. (**A**) T-DNA insertion sites in *atlbr-1, atlbr-2-1,* and *atlbr-2-2* with exons shown as black boxes. RT-PCR primers were designed to amplify the region containing the positions of T-DNA insertion (arrowhead). The orange bar represents the qRT-PCR primer extension site. (**B**) RT-PCR analysis of *AtLBR-1*, *AtLBR-2,* and *Actin* (control) transcripts in WT, *atlbr-1*, *atlbr-2-1*, *atlbr-2-2*, and *atlbr-DKO* plants. Transcript levels of the *PR* genes were determined by qRT-PCR with cDNA generated from WT and mutants seedlings treated with 100 μg/ml pLPS (**C**) or 1 μM flg22 (**D**) for the indicated time. Mean expression values were calculated from the results of three independent experiments. Means ± standard errors are presented. Significant differences among means were determined by two-way ANOVA followed by *post hoc* Bonferroni tests compared to WT plants; *P < 0.05, **P < 0.01. ROS generation by leaves of WT and *atlbr* mutants after treatments with 10 μg/ml pLPS (**E**) or 0.1 μM flg22 (**F**) for the indicated time. As a control, WT and *fls2* (FLAGELLIN-SENSITIVE 2) mutant leaves were treated with water and flg22, respectively. Means ± standard deviations are presented (n = 5). Significant differences among means compared to WT plants were determined by two-way ANOVA followed by *post hoc* Bonferroni tests; *P < 0.01.

To elucidate the relationship between the biological function of AtLBRs and LPS-induced defence responses in plants, we obtained three AtLBR T-DNA insertion lines from the Arabidopsis Biological Resource Center (ABRC) (Fig. 4A). *SC815603* (*atlbr-1*) carries a T-DNA insert in *At1g04970*. *SALK_050219* (*atlbr-2-1*) and *SALK_132326* (*atlbr-2-2*) carries a T-DNA insert in *At3g20270*. T-DNA insertion abolished expression of the *AtLBR* mRNA when examined by semiquantitative RT-PCR with appropriate primers (Fig. 4B, see Supplementary Table S1). We also generated a *atlbr-1/-2-1* double mutant (*atlbr-DKO*) by crossing *atlbr-1* and *atlbr-2-1*.

Since previous studies reported that LPS treatment induces some *PR* gene expression in *Arabidopsis*^1^,^30^,^31^, we first examined *PR1*, *PR2*, *PR3*, *PR4*, and *PR5* expressio in wild-type (WT) *Arabidopsis* by qRT-PCR. pLPS treatment induced expression of *PR1*, *PR4*, and *PR5*, but not of *PR2* and *PR3* in WT (data not shown). Thus, we next investigated *PR1*, *PR4*, and *PR5* expression by qRT-PCR in pLPS-treated *atlbr* mutants. Interestingly, we could detect *PR1* expression in WT but not in *atlbr* mutants at 24 h after pLPS treatments (Fig. 4C). However, at later time points of 48 and 72 h, both WT and *atlbr* mutants expressed the *PR1* gene at the same level (Fig. 4C, see Supplementary Fig. S1). Thus, *atlbr* mutants exhibit a significant delay of *PR1* expression. By using another *PR1* primer set, we also confirmed the delay of *PR1* gene expression in *atlbr* mutants (see Supplementary Fig. S2). In addition, to exclude the possibility of contamination by other bacterial components, we purified commercial pLPS and treated the seedlings with it. The purified pLPS also could induce *PR1* gene expression in WT, while that was defect in *atlbr* mutants (see Supplementary Fig. S3). This result excluded the possibility that this phenomenon is caused by contamination. As shown in Fig. 4C, we also found significant differences in *PR4* expression between WT and the two mutants, *atlbr-2-1* and *atlbr-DKO*, at 48 h after pLPS treatment. More research focusing specifically on the phenomenon is needed; however, since no difference was observed in *atlbr-2-2*, we tentatively concluded that this phenomenon might not be related to AtLBR-2 function. Thus, the deficiency of pLPS-induced *PR* gene expression in *atlbr* mutants is restricted only to *PR1* gene expression. As shown in Fig. 4D, both WT and mutant seedlings similarly induced the expression of *PR1* in response to 1 μM flg22, the bacterial flagellin peptide. This result demonstrates that AtLBR-mediated *PR1* induction is LPS specific. Further, we tested ROS generation as an early response to pLPS (Fig. 4E). Although ROS generations were induced equally well by flg22 treatments with both WT and mutants (Fig. 4F), those induced by pLPS treatments were significantly reduced in mutants. These results indicate that AtLBRs play important roles in LPS-induced *PR1* expression and ROS production, and suggest that AtLBRs play roles similar to those of hLBP, whose LPS binding facilitates LPS responses.

## Discussion

Although LBP/BPI/PLUNC superfamily proteins have been identified in various animals including non-mammalian vertebrates and invertebrates^10^,^11^,^12^,^13^,^14^,^15^, those of plants have not been defined. In this study, we characterised *Arabidopsis* LBP/BPI subfamily proteins termed AtLBR-1 and AtLBR-2 by analysing LPS-binding activity and studying the immune response to LPS in plants for the first time.

Phylogenetic analyses suggest that *Arabidopsis* AtLBRs are distantly related to mammalian LBP/BPI. However, we demonstrated that AtLBRs appear to have an immunological role similar to LBP/BPI subfamily proteins, including mammalian LBP, BPI, and Cg-BPIs, and PLUNC-related protein OCX-36. This result suggest that the LBP/BPI subfamily proteins with an the immunological role are distributed not only in animals but also in plants. However, the LBP/BPI subfamily also includes two lipid-binding proteins which have roles other than an immunological one; phospholipid transfer protein (PLTP) and cholesteryl ester transfer protein (CETP) play pivotal roles in LDL and HDL metabolism^7^,^25^,^26^, although they have been found only in vertebrates. This finding suggests that LBP/BPI proteins with the lipid-transfer role would have emerged after vertebrates diverged from a common ancestor. It is important to note that PLTP also shows an immunological role for LPS; it can bind and neutralise LPS^27^. Based on these observations and phylogenetic analyses (Fig. 1B) the following hypothesis can be proposed: a common ancestor of the LBP/BPI/PLUNC superfamily first played an immunological role, then some descendants acquired functions other than immunological ones (i.e., CETP and PLTP). On the other hand, we cannot exclude a second hypothesis: a common ancestor of the LBP/BPI/PLUNC superfamily first played a lipid-binding role, then its descendants were divided into two major subfamilies: proteins that acquired an immunological role (i.e., LBP, BPI, Cg-BPIs, OCX-36, and AtLBRs) and proteins with a role similar to the original one (i.e., PLTP and CETP).

Crystal structure analyses of LBP, BPI, and CETP reveal that each of the N- and C-terminal domains contains a single hydrophobic pocket with a bound phospholipid molecule^18^,^28^,^29^. Furthermore, CETP has two distinct hydrophobic tunnel openings in each domain, which is capped by one phospholipid^29^. Therefore, AtLBRs are also considered to have the hydrophobic pockets in their N- and C-terminal domains. Based on this finding and the result that N-terminal domains of AtLBRs bind to LPS, the N-terminal hydrophobic pocket may contribute to LPS binding. In addition, given that some LBP/BPI/PLUNC superfamily proteins bind to phospholipids via the hydrophobic pockets, AtLBRs may bind not only to LPS but also to other phospholipid molecules via these hydrophobic pockets.

In this study, we demonstrated the direct binding between AtLBRs and LPS. LPS consists of three distinct structural regions: O-antigen, oligosaccharide core, and lipid A. Although only the lipid A moiety is required for the activation of an immune response in animals, both the lipid A moiety and the O-antigen are important for a response in plants. A previous study revealed that treatment with lipid A induces *PR1* gene expression and NO and ROS production in *Arabidopsis*^1^,^4^,^30^. However, synthetic oligorhamnans, a common component of the otherwise highly variable O-antigen in LPS, also can trigger defence responses in *Arabidopsis*^31^. In this study, we found that the recombinant AtLBRs could bind to rough type LPS, which lacks O-antigen. This result suggests that O-antigen is not necessary for LPS recognition by AtLBRs. Thus, AtLBRs may affect the recognition of the lipid A moiety to regulate immune responses in plants. However, although the interaction of hBPI with smooth or rough type LPS is not significantly different^22^, AtLBRs showed higher binding affinity to rough type than to smooth type, suggesting that the O-antigen might prevent the binding of AtLBRs to the lipid A moiety of LPS.

Analysis of subcellular localisation showed that, in particular, AtLBR-2 was located in the apoplastic region. In addition, LPS-treated *atlbr* mutants showed significant decreases in *PR1* gene expression compared to that in the WT. These findings support the hypothesis that AtLBR-2 binds to LPS and catalyses LPS-induced immune responses in apoplastic space. Thus, AtLBR-2 may have activity similar to LBP rather than to BPI. Thus, similar to mammalian LBP which facilitates transfer of LPS to the TLR4/MD-2 receptor complex to amplify the immune responses against LPS, AtLBR-2 might also facilitate the transfer of LPS to a plant LPS receptor, with LORE being one recently identified candidate^4^. In contrast, although recombinant AtLBR-1N showed LPS-binding activity and *atlbr-1* is defective in LPS-induced *PR1* up-regulation, LPS treatment had not effect on *AtLBR-1* expression and we did not obtain clear localisation of the AtLBR-1-sfGFP fusion protein in the apoplastic region. We predict that AtLBR-1 may bind to LPS molecules and is involved in LPS signalling in intracellular regions such as the cytosol or vacuoles. More research is needed to better understand the relationship between AtLBR-1 and the LPS recognition mechanism in plants.

We demonstrated the deficiencies of LPS-induced *PR1* gene expression in *atlbr* mutants. However, the deficiencies restricted only expression of *PR1* but not *PR4* or *PR5*. It is well known that *PR* gene expression occurs after PAMP-induced salicylic acid (SA) accumulation^32^. SA is an important signal molecule in plant defence. Downstream of SA, *PR* gene induction occurs via two mechanisms: NON-EXPRESSOR OF PR1 (NPR1)-dependent and -independent pathways^32^. NPR1, a master regulator of SA-mediated defence genes, interacts with basal and systemic acquired resistance in plants. In the NPR1-dependent SA-induced pathway, *PR1* gene expression wes observed. In contrast, in the NPR1-independent SA-induced pathway, expression of *PR2* and *PR5* genes was observed^33^. These findings led to the hypothesis that AtLBRs are involved in the NPR1-dependent SA-induced pathway. In *atlbr* mutants, we detected the expression of *PR4*, the marker for ethylene signalling, suggesting that *atlbr* mutations had no effect on LPS-induced ethylene signalling.

It has been reported that SA signalling is preceded by ROS bursts mediated by NADPH oxidases and extracellular peroxidases^34^. ROS signals are involved in both upstream and downstream SA signalling in response to biotic and abiotic stress, including PAMPs, ozone, and UV-B treatment^34^. In this study, we showed that ROS generation significantly differed between pLPS-treated WT and *atlbr* mutants. The relatively low level of ROS generation in *atlbr* mutants may be one of the causes for deficiencies in *PR1* expression.

This study is the first to demonstrate that AtLBRs share functional similarities with the other LBP/BPI subfamily proteins. Our results suggest that plants have mechanistic parallels with animals for LPS perception. Our study may provide increased understanding of the details of LPS recognition mechanisms in plants and of the evolutionary relationship of the LPS perception system between plants and animals.

## Materials and Methods

### Elicitors

Rough and smooth LPS from *E. coli* were prepared from the Origami B strain (DE3) (Novagen) and the ATCC 25922 strain, respectively, as reported previously^19^. LPS from *Pseudomonas aeruginosa* serotype 10 was purchased from Sigma-Aldrich. The elicitor peptide flg22 was synthesised by Biologica Co.

### Sequence analysis

Protein sequences were obtained from NCBI: AtLBR-1 (NM_100375), AtLBR-2 (NP_188662), hLBP (CAA67226), and hBPI (ABD66755). Alignment was performed with Clustal-W implemented in BioEdit version 7. 0. 8. 0. (www.mbio.ncsu.edu/bioedit/bioedit.html). Conserved domain analyses were carried out using Conserved Domain Databases (http://www.ncbi.nlm.nih.gov/cdd/).

### cDNA cloning and plasmid construction

In the construction of the vector for protein expression in *E. coli*, RNA was extracted from *Arabidopsis* using the plant total RNA extraction Miniprep system (Viogene). DNase-treated RNA (3.75 μg) was reverse-transcribed using random primers (TAKARA BIO) and Superscript II reverse transcriptase (Invitrogen) according to the manufacturer’s protocol. cDNA fragments for N-terminal AtLBR-1 (AtLBR-1N) coding 215 amino acid residues (1–215) were amplified by PCR using PrimeSTAR GXL DNA Polymerase (TAKARA BIO). Amplification was first carried out with nested primer set Nes1n, followed by primer set ATLBR-1n (see Supplementary Table S1). cDNA fragments for N-terminal AtLBR-2 (AtLBR-2N) coding 214 amino acid residues (1–214) were also created as described above using nested primer set Nes2n and primer set ATLBR-2n (see Supplementary Table S1). For expression as a N-terminal thioredoxin (Trx) and His-tag fusion protein, these fragments were also amplified using primer set pSU2amp (see Supplementary Table S1), then they were cloned into pET32a(+) by yeast recombinational cloning using pYES2/CT as a helper plasmid^35^. The nucleotide sequences of these recombinant proteins were confirmed by sequencing using an ABI 310 genetic analyser (Applied Biosystems). A fusion protein of Trx with His-tags was used as a control in this study. For the subcellular localisation analysis, we cloned cDNA of full-length AtLBR-1 and the genomic sequence of AtLBR-2 into P35S-sfGFP-TNos vector^36^ by yeast homologous recombination^37^.

### Protein expression and purification

Protein expression and purification were preformed based on a method reported previously^19^. In brief, Origami B (DE3) was transformed with the plasmids and cultured. Extraction was performed using a French Pressure Cell Press (Otake Seisakusho). The supernatants were applied to a nickel-absorbed chelating sepharose column. After washing with the buffer, including 50 mM imidazole with or without 0.5% Triton X-100 (TX-100), adsorbed proteins were eluted with the same buffer, changing the concentration of imidazole to 500 mM. The protein preparations were analysed by SDS-PAGE (12% acrylamide gel) and Coomassie brilliant blue (CBB) staining. Detection of LPS was performed by immunoblotting (18% acrylamide gel) using an anti-LPS monoclonal antibody NW1 222-5 (Hycult Biotechnology), alkaline phosphatase-conjugated anti-mouse Ig (American Qualex), and BCIP/NBT Colour Development Substrate (Promega)^19^.

### LPS-binding assay and competition test

This procedure is based on a method published previously^19^. LPS-free recombinant proteins were prepared by affinity chromatography on Ni-columns using a washing buffer containing 0.5% TX-100. Recombinant proteins were diluted to 10 μg/ml in a buffer (50 mM phosphate buffer, pH 7.0, 150 mM NaCl, 50 mM imidazole, 1 mM PMSF, 5 mM 2-ME, and 1 mg/ml of BSA) and incubated at room temperature for 30 min with 10 μg/ml LPS from *E. coli* (smooth type). In the competition test, recombinant proteins were incubated with 10 μg/ml smooth type LPS from *E. coli* with or without 100 μg/ml LPS from *P. aeruginosa*. The mixtures were applied to His Spin Trap columns (GE Healthcare), washed, and eluted according to the manufacturer’s protocol by using the same buffers used in the column chromatography. Without dilution, eluted samples were analysed to determine LPS content by immunoblotting.

### Subcellular localisation analysis

An appropriate plasmid mix for expression of sfGFP fusion and DsRed-monomer proteins was co-bombarded with gold particles by the biolistic particle delivery system (PDS-1000/He, Bio-Rad) into onion (*Allium cepa*) epidermal cell layers. The DsRed-monomer was used as a cytosolic marker protein. After incubation at 23 °C for 16 h in darkness, fluorescence images were obtained with a confocal microscope (TCS SP8, Leica). A white light laser was used for the excitation lights (488 and 556 nm for sfGFP and DsRed-monomer, respectively). The emission signals were captured at 498–549 nm for sfGFP and at 566–650 nm for DsRed-monomer. To induce plasmolysis, the onion epidermal cell layers were treated with 20% sucrose solution for 10 min before the microscopic observation.

### Plant material and growth conditions

*Arabidopsis* ecotype Col-0 was used as a control in this study. T-DNA insertion line CS815603 (*atlbr-1*) was provided by SAIL (Syngenta *Arabidopsis* Insertion Library). T-DNA insertion lines SALK_050219 (*atlbr-2-1*) and SALK_132326 (*atlbr-2-2*) were provided by SIGnAL (Salk Institute Genomic Analysis Laboratory). All plants were in the Col background. *atlbr-DKO* double knockout mutants were obtained by crossing *atlbr-1* and *atlbr-2-1* mutants. AtLBR-1-, AtLBR-2- and T-DNA-specific primers were used to select plants homozygous for the insert. All plant were grown at 22–24 °C with a 16 h light/−8 h dark cycle.

### Elicitor treatment and real-time PCR (qRT-PCR) analysis

After seedlings were grown for 5 days on MS agar plates under the conditions defined above, they were transferred to liquid MS medium supplied with the indicated elicitor preparations under continuous light conditions. After elicitor treatment, whole seedlings were ground to a powder in liquid nitrogen. RNA was extracted and reverse transcribed using 1 μg of total RNA. qRT-PCR was run on a PikoReal real-time PCR system (Thermo Fisher Scientific) according to the manufacturer’s recommendations with the following conditions: 1 cycle of 1 min at 95 °C, and 40 cycles of 5 s at 95 °C and 30 s at 60 °C. *β-Tubulin4* was used as an internal standard. The gene-specific primers were used as described^38^ (see Supplementary Table S1). Each experiment was repeated at least three times.

### ROS measurements

ROS generation was determined using the H_2_O_2_-dependent chemiluminescence reaction based on a method reported previously^39^. *Arabidopsis* leaves of 3- to 4-week-old plants grown on MS agar plates were cut into 2 mm slices and floated on H_2_O overnight. Slices (20 mg fresh weight) were exposed to elicitor solutions, and 2 μl of the solution was transferred to assay tubes containing 0.1 ml of H_2_O supplemented with 20 μM luminol and 1 μg horseradish peroxidase (Sigma). Luminescence was measured in a GloMax 20/20 Luminometer (Promega) for 28 min after treatment.

## Additional Information

**How to cite this article**: Iizasa, S. *et al.* *Arabidopsis* LBP/BPI related-1 and -2 bind to LPS directly and regulate *PR1* expression. *Sci. Rep.* **6**, 27527; doi: 10.1038/srep27527 (2016).

## Supplementary Material

Supplementary Information
