# Supplementary material for: Arabidopsis LBP/BPI related-1 and -2 bind to LPS directly and regulate PR1 expression
Source: Sci Rep. 2016 Jun 8;6:27527. doi: 10.1038/srep27527 (PMC4897604; doi:10.1038/srep27527)
Supplement: Supplementary Information [file srep27527-s1.pdf]

***Arabidopsis* LBP/BPI related-1 and -2 bind to LPS directly and regulate *PR1* expression**

Sayaka Iizasa*[1, 2], Ei'ichi Iizasa[3], Sawako Matsuzaki[1, 4], Hiroyuki Tanaka[5], Yutaka Kodama[5], Keiichi Watanabe[2, 4], and Yukio Nagano*[1, 2]

[1]Analytical Research Center for Experimental Sciences, Saga University, 1 Honjo-machi, Saga 840-8502, Japan. [2]Department of Biological Science and Technology, The United Graduate School of Agricultural Sciences, Kagoshima University, 1-21-24 Korimoto, Kagoshima 890-8580, Japan. [3]Department of Immunology, Graduate School of Medical and Dental Sciences, Kagoshima University, 8-35-1 Sakuragaoka, Kagoshima 890-8544, Japan. [4]Department of Applied Biochemistry and Food Science, Faculty of Agriculture, Saga University, 1 Honjo-machi, Saga 840-8502, Japan. [5]Center for Bioscience Research and Education, Utsunomiya University, Tochigi 321-8505, Japan. Correspondence and requests for materials should be addressed to I. S. (k8930172@kadai.jp) or N. Y. (nagano@cc.saga-u.ac.jp)

Supplemental Table S1. Primers set used in this study.

For AtLBRs cloning and plasmid construction.

Set	F/R	Sequence
ATLBR-1n	F	5′-TTCGAGCTCCGTCGAACCGATTCATTCACATCGGT-3′
	R	5′-AGCAGCCGGATCTCATTATATAGGATCACTGGTGA-3′
ATLBR-2n	F	5′-TTCGAGCTCCGTCGAAACAATGGCGGTCACATTTC-3′
	R	5′-AGCAGCCGGATCTCATTAGACAGGGTTGCCTGTAA-3′
Nes1n	F	5′-CCAAGTTTCTTCTTCTTGCC-3′
	R	5′-GTGAAAACTCTAACTACCGA-3′
Nes2n	F	5′-TCTTCGTCTCGGTGTCATCG-3’
	R	5′-GCCAACTTCAACTGTAGTTA-3′
pSU2amp	F	5′-GGCCATGGCTGATATCGGATCCGAATTCGAGCTCCGTCGA-3′
	R	5′-ACTCAGCTTCCTTTCGGGCTTTGTTAGCAGCCGGATCTCA-3′

For semiquantitative RT-PCR.

Set	F/R	Sequence
RT-AtLBR1	F	5′-GCACCGATTCATTCACATCG-3′
	R	5′-CCTTTGGAAGGCTTTGTAGG-3′
RT-AtLBR2	F	5′-GGTGGTTGATGCATTTCAAA-3′
	R	5′-CTTCAGGCTTACGTACATGC -3′
RT-Actin	F	5′-TCTTGATCTTGCTGGTCGTG-3′
	R	5′-GAGCTGGTTTTGGCTGTCTC-3′

For quantitative RT-PCR (qRT-PCR).

Set	F/R	Sequence
qAtLBR-1	F	5′- CCATTGAGTTGGAAGGAGGA-3′
	R	5′- TGGCAATGGTACTTTCCACA-3′
qAtLBR-2	F	5′- CGGGTCCATTCTAAGCACAT-3′
	R	5′- ATCGTCGCATCAATTCCATT-3′
qPR1	F	5′- GGAGCTACGCAGAACAACTAAGA-3′
	R	5′- CCCACGAGGATCATAGTTGCAACTGA-3′
qPR4	F	5′- TTGCTCCACGTGGGATGCTGAT-3′
	R	5′- AGCTCATTGCCACAGTCGACAA-3′
qPR5	F	5′- CGGTACAAGTGAAGGTGCTCGTT-3′
	R	5′- GCCTCGTAGATGGTTACAATGTCA-3′
qβ-Tubulin4	F	5′- GAGGGAGCCATTGACAACATCTT-3′
	R	5′- GCGAACAGTTCACAGCTATGTTCA-3′

For Supplementary Fig. S1 (another set of *PR1* primers).

Set	F/R	Sequence
qPR1-2	F	5′- GTCTTTGTAGCTCTTGTAGGTG-3′
	R	5′- CAACCCTCTCGTCCCACTGCAT-3′

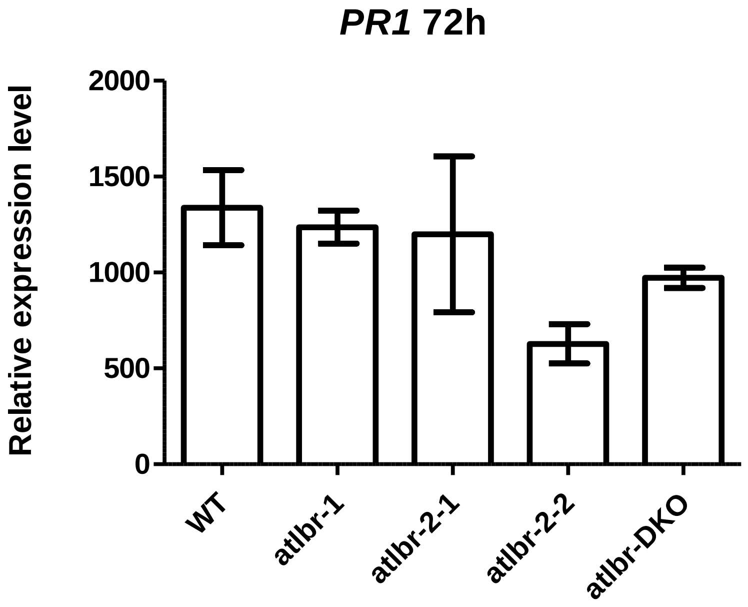

Supplementary Fig. S1. *PR1* mRNA levels in WT and *atlbr* mutant *Arabidopsis* seedlings treated with 100 µg/ml pLPS during 72 h were detected by qRT-PCR. Mean expression values were calculated from the results of three independent experiments. Means ± standard errors are presented. Significant differences among means compared with WT plants were evaluated by one-way ANOVA followed by Tukey's multiple comparison test.

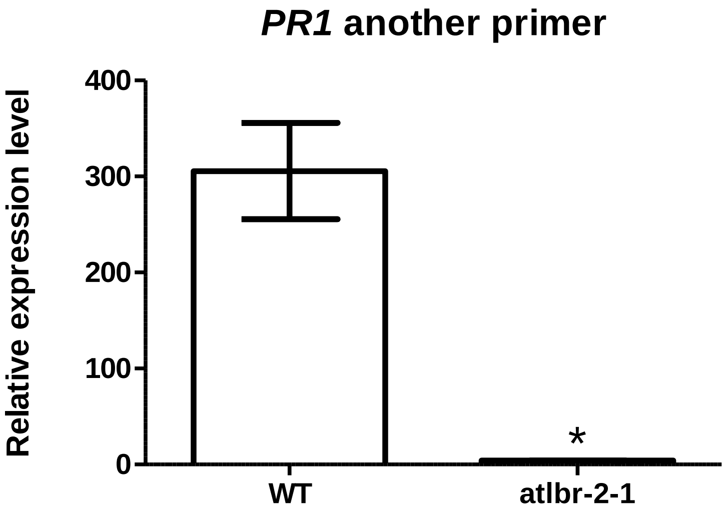

Supplementary Fig. S2. *PR1* mRNA levels in WT and *atlbr-2 Arabidopsis* seedlings treated with 100 μg/ml pLPS during 24 h were detected by qRT-PCR using another set of primers (Supplementary Table S1). Mean expression values were calculated from the results of three independent experiments. Means ± standard errors are presented. The significant difference between means was evaluated with a *t*-test; $*P < 0.05$.

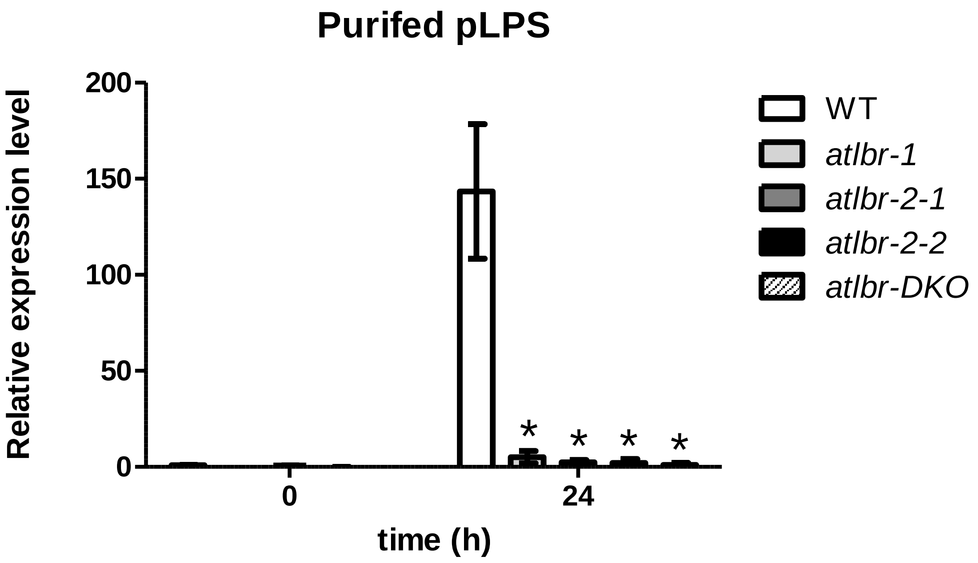

Supplementary Fig. S3. Purchased *P. aeruginosa* LPS was purified by degradation of nucleic acids and proteins with DNase I, RNase, and proteinase K. *PR1* mRNA levels in WT and *atlbr* mutant *Arabidopsis* seedlings treated with 100 µg/ml purified pLPS during 24 h were detected by qRT-PCR. Mean expression values were calculated from the results of three independent experiments. Means ± standard errors are presented. Significant differences among means compared to WT plants were evaluated by two-way ANOVA followed by *post hoc* Bonferroni tests; $*P < 0.001$.
